# Training in quality improvement for the next generation of psychiatrists

**DOI:** 10.1192/pb.bp.115.051409

**Published:** 2017-02

**Authors:** Elizabeth Ewins, Rob Macpherson, Geoff van der Linden, Stephen Arnott

**Affiliations:** 1 Avon and Wiltshire Mental Health Partnership NHS Trust; 2 Health Education South West, Bristol

## Abstract

Quality improvement (QI) projects have been shown to positively influence patient care. They provide opportunities for trainees to present and publish their work locally and nationally, and to gain valuable leadership and management experience. We describe a pilot project to engage in QI trainees across a National Health Service trust and a school of psychiatry. After the first year of this programme over half of psychiatry trainees in the school (58% of core trainees and 47% of advanced trainees) are participating in 28 individual QI projects and QI project methodology is to become embedded in the core psychiatry training course. Specialty doctors, consultants, foundation doctors, general practitioner trainees, medical students and the wider multidisciplinary team have all become engaged alongside trainees, working with patients and their families to identify problems to tackle and ideas to test.

Foundation doctors and core medical trainees are being trained to undertake quality improvement (QI) projects and are doing so competently and effectively.^1,2^ QI projects have been embedded in the Foundation Programme curriculum,^3^ with an expectation that trainees plan, implement, complete and present a QI project as part of their training, using the results to improve patient care. QI projects have been widely adopted across medicine and are in many areas superseding traditional audit as a way to develop services. However, the development of this methodology has been slower in the mental health field and QI projects are new to most psychiatrists. Here we outline and summarise in simple steps how to undertake a QI project. We also describe our cross-deanery project led in Avon and Wiltshire Mental Health Partnership NHS Trust and the Severn School of Psychiatry, which aims to train and support psychiatrists of the future to become actively engaged in QI projects.

## What is a QI project?

QI projects aim to improve patient safety, treatment effectiveness and efficiency, and the patient experience. They are real-time, dynamic processes involving focused, progressive, small-scale changes through a simple structured framework, which enable visible and effective change over a short period of time. QI projects are becoming increasingly important because of the limited resources available in the National Health Service (NHS). They can support service change and to achieve this may engage all professional groups, including trainees. Increasingly, evidence of QI is becoming a training requirement and is taking the place of audit as a subject to discuss at trainee reviews and job interviews.

**Fig. 1:**
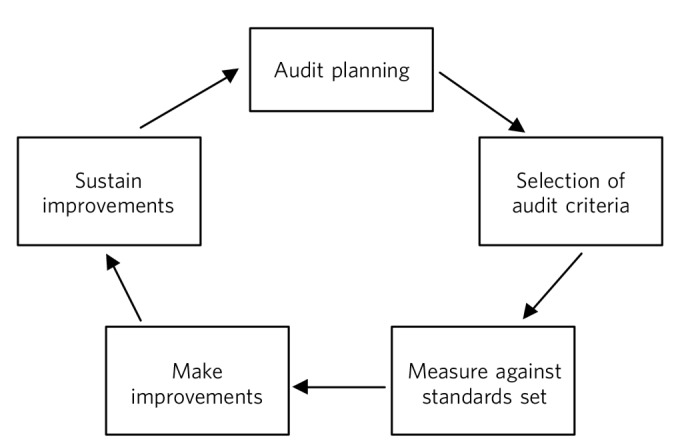
Representation of the traditional audit cycle (based on Benjamin^8^).

Often the relationship between audit and QI projects is not clearly understood. Audit is also generally about quality improvement, beginning with identifying an audit subject, selecting audit standards and measuring the level of performance, then making improvements and re-auditing (Fig. 1). The focus is therefore on data collection.^4^ This area of practice has been criticised, as often the audit cycle has been left incomplete and the improvement part of the cycle left neglected.^5,6^ QI projects are advantageously placed as they can be seen as part of the improvement element of the audit cycle, where the focus of the project is explicitly on making a service improvement instead of collecting data.^7^ Although more complex than an audit, QI projects focus on changing complex adaptive systems and can empower doctors to investigate problems, identify solutions and work within a team to raise standards.

## Practical steps to carrying out a QI project

The first step in developing a QI project is identifying a specific aspect of clinical practice that could be improved. This may be something that has been highlighted by an audit or identified by the wider team. It may be aligned to the local trust's quality and safety agenda, something that has been identified as a clinical incident or near-miss, or raised by patients or their families as a problem or idea to test. Finally, it may simply be an area recognised by local clinicians as something which is time consuming or frustrating and which could be improved. The project has a small focus initially, so rather than looking at 100 patient notes one might begin with just a single patient, or instead of trying to improve a whole hospital the focus may be on a single ward. Once an improvement has been proven to work on a small scale, it can be then tested on another patient or another ward, gradually being systematically scaled up and spread to become embedded in an entire hospital or trust. Depending on complexity, the projects can be undertaken within a 6-month training post.

The Institute for Healthcare Improvement recommend asking three questions^9^ based on the ‘model for improvement’ when planning a project:

1. What are you trying to accomplish? This helps to set the aim of the project, which should be SMART (specific, measurable, achievable, realistic and timely).
2. How will you know that a change is an improvement? This helps to think about what can be measured to illustrate the impact of the change. What would be an easy measurement? This needs to be done at baseline and then repeated at regular intervals so that the change can lead to learning and to show that it works.
3. What changes can we make that will result in improvement? Possible ideas of changes to implement to make an improvement can be brainstormed. The current sequence of events already used can be examined and areas for improvement identified, for example by eliminating unnecessary tasks or steps, clarifying roles within the process, or by reducing delays and duplication.

### Testing changes: the ‘plan, do, study, act’ (PDSA) cycle

**Fig. 2:**
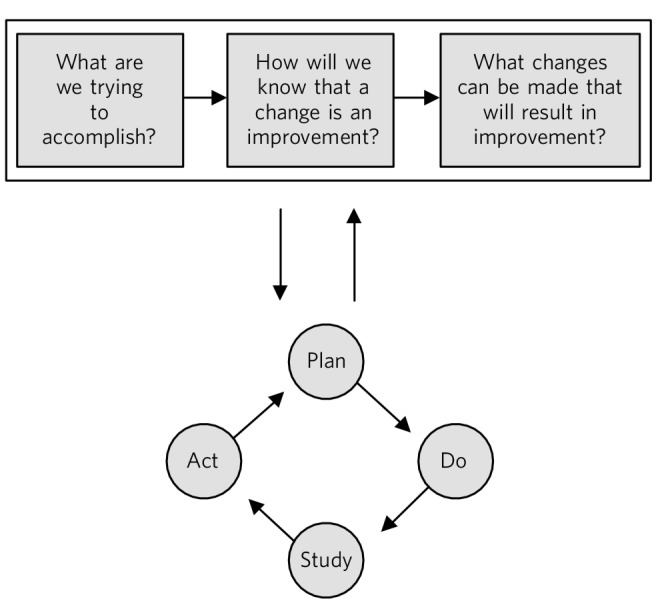
The model for improvement is used as a framework to structure a quality improvement project (it includes going through several ‘plan, do, study, act’ (PDSA) cycles).^9^ Based on Langley et al.^11^

The ‘plan, do, study, act’ (PDSA) cycles can be used as a way to develop, test and then implement a change on a small scale and in a real work setting (Fig. 2).^9,10^ Multiple PDSA cycles will be required to fully implement a QI project.

#### Stage 1: plan

The first stage is about planning a test of change. This involves taking a single idea and making a prediction as to what might happen when the change is implemented. A test and way of measuring whether what actually happens meets that prediction needs to be designed so that the impact of the change can be evaluated and learned from. This might be numerical data, such as increasing numbers of reviews of patients, or reducing numbers of critical incidents, and may also include patient and/or staff satisfaction scores or comments. Once you have determined what you are going to measure, make a baseline measurement. Determine what target you are aiming for: is it realistic? Plan the time you have available: for example, do you want to complete the project within the time frame of a training post? The planning stage can take time, but good planning will ensure a more successful project.

**Fig. 3:**
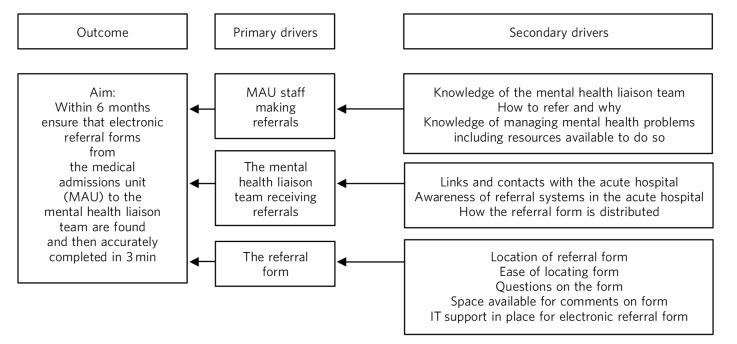
A driver diagram showing primary and secondary drivers for a quality improvement project trying to improve the time taken for referrals to be made to a psychiatry liaison service. From this, ideas for change can be generated, such as producing a short guide of how to complete the referral form, providing a short training session for staff, or ensuring referral forms are located with other referral forms in the hospital. As well as measuring the time taken for referral forms to be completed at regular intervals to assess the impact of the change, staff satisfaction scores and qualitative data could also be gathered.

A useful tool for the early stages of planning a QI project is a driver diagram (Fig. 3), which can help to identify what steps could be taken to make an improvement. This has three columns: outcome (the aim of the project) and primary and secondary drivers. Primary drivers are the overall improvement areas that need to be addressed to achieve the desired outcome. Secondary drivers are the specific areas where changes or interventions can be made, motivated by the primary drivers.

#### Stage 2: do

Following careful planning, one small area of change can be identified. The second stage of the PDSA cycle is where the change is actually implemented. Measures of the impact of change should be taken from an early point and frequently to monitor the effect of the change. Any problems or unexpected results are noted while the change is carried out.

#### Stage 3: study

**Fig. 4:**
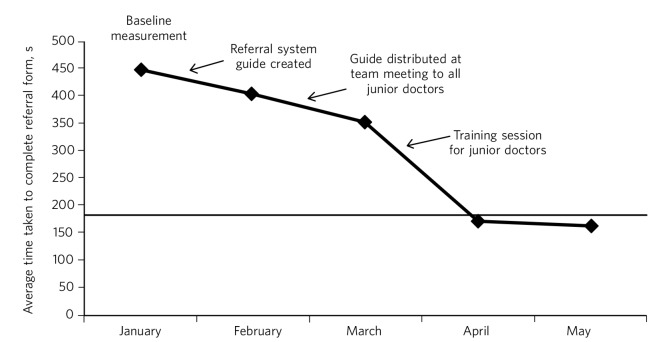
Plotting results for the example quality improvement project in Fig. 3. The time taken to complete referral forms was measured by timing eight junior doctors; each point on the graph shows the average of these measurements. A goal line (of 180 s) is shown and interventions made at each ‘plan, do, study, act’ (PDSA) cycle are labelled on the graph so the effect of each change can be clearly seen.

The third stage involves analysing collected data and comparing that with the predictions made. A graphical representation of the measurements taken can be a useful way of plotting results to illustrate the pattern observed as changes are made.^12^ A goal line can be shown so one can see at a glance where the work is in relation to achieving the aim. Changes or improvements that are made (in repeated PDSA cycles) can annotate the graph to clearly demonstrate the impact of those changes (Fig. 4).

#### Stage 4: act

In the final stage of a PDSA cycle you can ask whether the change you made achieved your aim. If not, what modifications to the change could be made? Or what other ideas can be put in place? When you are ready to make another change, the next PDSA cycle can be outlined.

### Implementing and spreading changes

By going through several PDSA cycles a small change can be tested and refined. Once a successful improvement has been proven on a small scale, it can then be tested on a larger scale. This might be across several wards or across several community teams. Once these pilot changes are proven to be successful, they can be embedded in everyday practice and incorporated into local trust policy.

## Training in QI for the next generation of psychiatrists – a pilot programme

This is the first year of a pilot developed in the Severn Deanery to support core and advanced trainees to become involved in QI. The programme is being led by an advanced trainee (E.E.) as part of an Educational Fellowship awarded by the Deanery.

Until 2015 it has been a deanery requirement of trainees to carry out an audit project annually. This was amended so that all trainees are to be involved in either an audit or a QI project each year, and if an audit is undertaken trainees are encouraged to use QI methodology for the implementation of change part of the cycle. The structure of training and supporting trainees to undertake their own QI projects was adapted from the local Foundation School^1^ and the Royal College of Physician's ‘Learning to make a difference’ programme.^2^ A half-day training session was developed to teach trainees about QI and project methodology, including using PDSA cycles in practical examples, so that they could undertake their own projects. This was delivered by E.E. alongside the local trust audit and QI department lead. Trainees were encouraged to come up with their own ideas for projects and to work with other trainees to implement changes, under the supervision of a higher trainee, specialty doctor or consultant. They were supported in including the wider multidisciplinary team in developing and implementing projects, as it was felt that this would be more likely to lead to successful and sustainable changes being made.

Training was initially targeted at trainees, but a growing number of specialist doctors and consultants requested to attend and they were invited to a second training session. A resource handbook was developed and a series of short follow-up sessions put in place to provide further advice about QI methodology and to help support trainees' projects. Trainees were asked to register their projects by completing a short online form on the local trust audit and QI academy webpage so the trust can monitor participation.

### Results so far

In the first year of this programme, QI projects (28 in total) are being undertaken by 58% of core trainees (21 of 36) and 47% of advanced trainees (16 of 34). A growing number of specialty doctors (6 of 54; 11% of the Trust's total), consultants (24 of 111; 22%) and colleagues from the wider multidisciplinary team, as well as foundation doctors and medical students, are becoming involved in projects. Examples of current projects being undertaken by trainees in the Deanery are shown in Box 1. Some trainees have chosen to undertake educational QI projects, which are being used to improve training and trainee representation in their organisations and are felt to be a way of engaging trainees in the trust.

Evaluation of the programme so far through post-course questionnaires and semi-structured interviews held at the end of projects has shown globally positive feedback from consultants and high engagement and enjoyment from trainees. Trainees report high satisfaction owing to being able to choose their own QI projects rather than being instructed what to do. All trainees report that training sessions and follow-up advice has been helpful, and feel that this should be provided to all staff across the multidisciplinary team. One trainee fed back that ‘the quality improvements often end up involving other team members so it would be good to get them on side’, whereas another trainee highlighted that ‘training is needed for senior staff members who we may need to get on board.’ Additionally, trainees reflected that most projects needed senior input to facilitate implementation and sustainability of successful changes, and several projects have needed advice on governance issues, which has been provided by the Trust's Quality Academy, responsible for audit and QI projects.

We have found that owing to the nature of our work in mental health, QI projects sometimes need to be structured in a different way than they would be in an acute hospital setting. Careful consideration is needed to find the most appropriate method of change measurement, as frequently qualitative data may be available and innovative methods of quantitative data have been required. We have also found that QI projects have often had to run over a longer period than they perhaps would in an acute hospital, perhaps owing to the longer in-patient stay in a psychiatric hospital.

During the pilot we found that forming close links with the trust Quality Academy provided invaluable assistance in setting up the scheme. The Academy has a QI project lead, who provides advice and training for trainees; they have been particularly helpful with guidance regarding governance and what permissions might need to be sought. Support from trust medical management leads, including the medical director and director of medical education, has also been key.

As well as positively influencing patient care, trainees report that projects are providing them with invaluable opportunities for leadership and management experience. One advanced trainee leading a QI project reported they had gained ‘experience of leading a team as well as networking with other teams, management experience through attending meetings and presenting ideas, plus the project has provided opportunities to present at a departmental and regional level, as well as an opportunity for publication. This is in addition to positively influencing the future of mental health services’. Many trainees are beginning to present and publish their work and we encourage trainees to do so even if a project has not been fully successful, as much will have been learned by the trainees, and can be learned by the Trust, from all projects.

**Box 1** Quality improvement projects being undertaken by trainees across the Severn Deanery

- Improving handover between trainees
- Ensuring physical health monitoring of patients prescribed antipsychotics
- Improving the quality of letters written to general practitioners
- Ensuring timely access to radiology results
- Promoting awareness of mental health in an acute hospital
- Providing support for new consultants
- Advance care planning in later life
- Improving the quality of ward rounds in forensic services
- Training nursing staff about physical healthcare issues
- Providing patients and their families with information regarding child and adolescent mental health services (CAMHS)
- Improving the local academic programme
- Developing an out-of-hours handbook for trainees on call
- Ensuring physical health assessments for patients in early intervention in psychosis
- Improving access to mental health assessments for women during the antenatal period
- Improving trainee representation across the mental health trust

**Box 2** Useful resources

- The Institute for Healthcare Improvement website (www.ihi.org) provides many free resources to guide professionals through a quality improvement (QI) project (e.g. short videos which describe the steps involved).
- BMJ Quality (http://quality.bmj.com) has an online guide to implementing a QI project and then writing it up, producing a publishable paper as a result. It can be useful to buy a licence to do this and follow the steps (licences last for 1 year so in the case of longer-term projects it may be prudent to sign up later rather than at the start of a project). Note that demonstration of clear ‘plan, do, study, act’ (PDSA) cycles is required for successful publication. There is a growing database of published QI projects which may prove inspirational for ideas that can be developed in psychiatry.
- Local audit departments may be able to support projects directly and help identify potential QI project areas. Health Education England also publishes innovative ideas which can provide further inspiration (http://hee.nhs.uk/). Service user groups can be another source of ideas for QI projects.

### Future plans

The training course is to be incorporated into the Deanery core trainees' course and it is expected that all new core trainees who have joined the Deanery in the 2015 summer intake will participate in a QI project each year. Formal evaluation of the impact of training and QI projects is to be undertaken for this cohort. Those trainees who have successfully completed a QI project will be encouraged to become mentors and local QI leads in their area for future projects, providing sustainability for the projects as well as supervision, teaching and leadership experience for trainees.

QI projects undertaken by trainees and their seniors are to be regularly presented at the Trust's Medicines Advisory Group meetings, which will not only spread innovative ideas but further encourage psychiatrists to become involved in projects. Connections are being developed with service user groups and local patient safety programmes to help trainees define problems to tackle and ideas to test. Links have been made with the West of England Academic Health Science Network and the Royal College of Psychiatrists' South West Division E-volution programme (www.rcpsych.ac.uk/workinpsychiatry/divisions/southwest/innovationinthesouthwest.aspx) to promote the wider spread of quality improvement and innovation.

We have compiled a list of useful resources (Box 2) and tips for developing a QI project (Box 3) drawing on our own experience in the Trust.

**Box 3** Top tips for completing a quality improvement (QI) project

- **Take time to plan your project**. You may be keen to start putting in place changes and improvements, but ensuring careful planning will mean that a project is more likely to be successful.
- **Have a SMART aim**. Be clear and focused. Have a clear aim so that everyone knows what you are trying to achieve. Make your aim SMART (specific, measurable, achievable and agreed, relevant and time-bound).
- **Keep it small**. This will help ensure an improvement works. Once it is proven to work on a small scale, it can be rolled out more widely.
- **Think outside the box**. Get a group of interested trainees, consultants and the wider multidisciplinary team to think about the project with you and help brainstorm ideas for improvements. This will engage others and help the project to be a success. Get advice from your audit department.
- **Work in a team**. This is more fun, you will come up with more ideas and will feel more motivated.
- **Involve key stakeholders**. Who needs to know about the project for it to be a success? Do you need any approval to carry out changes? This might be a team consultant and team or ward manager, or it could include your clinical director, medical director or director of medical education or head of school. You could present your idea at a local academic meeting.
- **Sustainability**. Think about how you can make your improvements continue, especially if you are in a training post and will be moving on in 6 months or a year. You will need to involve your multidisciplinary team and local team managers.
- **Organise your time**. Think about how much time you have available to complete the project. Set yourself a time frame and stick to it. If you will be moving from a training post, think about whether you want to continue making changes after you leave or whether you need to do some succession planning.
- **Make the most of the opportunity**. After all that hard work, make sure you get some rewards! Present your work locally, submit a poster to a conference, and write up your work and get it published. QI projects frequently provide leadership and management experience, and often teaching opportunities which you can mention at your annual review of competence progression (ARCP), annual appraisals and at job interviews.
